# Conserved and differing functions of the endocrine system across different social systems – oxytocin as a case study

**DOI:** 10.3389/fendo.2024.1418089

**Published:** 2024-07-11

**Authors:** Meghan J. Sosnowski, Sarah F. Brosnan

**Affiliations:** ^1^ California National Primate Research Center, University of California, Davis, Davis, CA, United States; ^2^ Department of Psychology, University of California, Davis, Davis, CA, United States; ^3^ Department of Psychology, Georgia State University, Atlanta, GA, United States; ^4^ Language Research Center, Georgia State University, Decatur, GA, United States; ^5^ Center for Behavioral Neuroscience, Georgia State University, Atlanta, GA, United States; ^6^ Neuroscience Institute, Georgia State University, Atlanta, GA, United States

**Keywords:** oxytocin, endocrinology, non-traditional models, hormones, behavior, social systems

## Abstract

A key goal of the field of endocrinology has been to understand the hormonal mechanisms that drive social behavior and influence reactions to others, such as oxytocin. However, it has sometimes been challenging to understand which aspects and influences of hormonal action are conserved and common among mammalian species, and which effects differ based on features of these species, such as social system. This challenge has been exacerbated by a focus on a relatively small number of traditional model species. In this review, we first demonstrate the benefits of using non-traditional models for the study of hormones, with a focus on oxytocin as a case study in adding species with diverse social systems. We then expand our discussion to explore differing effects of oxytocin (and its response to behavior) within a species, with a particular focus on relationship context and social environment among primate species. Finally, we suggest key areas for future exploration of oxytocin’s action centrally and peripherally, and how non-traditional models can be an important resource for understanding the breadth of oxytocin’s potential effects.

## 1 Introduction

A key question in the study of any aspect of behavior, and the mechanisms underpinning it, is the degree to which systems are conserved - or diverge – across different social structures. One challenge, to this, of course, is determining which factors influence these similarities and differences. Given that differences in ecology, cognition, and other factors also vary among species with different social structures, making it difficult to disentangle how these relate to specific behaviors or traits, a convenient feature of comparative approaches to behavioral endocrinology is that at least some components of the endocrine system have been conserved across a wide variety of species. There is strong evidence for the conservation of many hormones and hormonal systems across species, even when social systems have diverged, providing researchers with an opportunity to narrow down on the basic function of these hormones while also exploring how these functions might be co-opted to increase fitness within each social system.

A particularly interesting hormone in this regard is oxytocin and its analogues. Oxytocin has been implicated in a wide variety of social behaviors and social systems, yet there is also variability in effects and even the directionality of those effects. Notably, research has not found consistent impacts on social behavior and cognition, despite significant research effort, and this is a key area of interest. On the one hand, oxytocin has demonstrated effects on social behavior, often increasing prosocial giving and social behaviors such as grooming. On the other, oxytocin can also increase aggression and protective behavior of kin and/or ingroup members, especially towards outgroup individuals, suggesting that rather than increasing prosocial behavior universally, it works differently on ingroup versus outgroup members. Perhaps more concerningly, an increasing number of studies find no effect of oxytocin, and results vary depending on whether we look at the effect of behavior on oxytocin or oxytocin on behavior, suggesting that we are missing some of the important subtleties of this interaction.

While these variations have led to important new hypotheses about the role of oxytocin and novel theoretical approaches to understanding how oxytocin works, especially on behavior and cognition, one major limitation is that despite the fact that oxytocin, or an analogue, seems to be important in most vertebrate species (and maybe beyond), most research has focused on a few model systems (particularly prairie voles, rhesus monkeys, and chimpanzees). Although these species have led to important insights, they differ in social structure, ecological niche, and phylogeny, making it difficult to pinpoint the effects of any one of these features. It is likely that we are missing important insights from other species that would, in particular, shed light on how social structure might interact with oxytocin. Thus, in this review we largely focus on non-traditional model systems with explicitly different social systems to consider what we know about and how we can further study oxytocin’s function in specific social contexts, as well as how that informs us about broad commonalities in oxytocin.

## 2 The exploration of oxytocin through different social systems

Oxytocin’s role in social cognition and behavior was first studied as a key component of maternal-offspring interaction and bonding. Oxytocin, a neurohypophyseal nonapeptide hormone, had previously been shown to play a key role in the parturition and lactation processes (1), and it was subsequently found to have effects on maternal behavior in rodent models. The role of oxytocin was established causally when oxytocin injection into the brain of virgin Sprague-Dawley rats (*Rattus norvegicus domestica*), which typically avoid rat pups, resulted in nurturing and maternal-like behavior toward pups within hours following injection (2). Although it is important to note that such results have not always replicated even among different strains within the species itself (3), subsequent studies have used oxytocin antagonists to show that endogenous oxytocin is necessary for the formation of maternal behavior for rats that have just given birth (4), and that deficits in oxytocin receptors (via oxytocin receptor knockout) result in consistent deficits in nurturing behavior (5), solidifying a necessary role of oxytocin for typical maternal behavior in this species.

Rats, however, only represent one species with one type of parental system - namely, a uniparental maternal model with no specific preference toward one’s own offspring. Female rats typically do not live in large groups, and instead maintain a solitary nest for their litter of pups; thus, there is little need to develop a preference for one’s own pups as opposed to strange pups, as it is unlikely that another rat’s pups will find their way into the nest. However, in group-living species, and species which give birth to only one or two offspring at a time, mothers benefit from preferentially mothering their own offspring over others – and through study of one such species, domestic sheep (*Ovis aries*), we have been able to explore oxytocin’s role in maternal-offspring bonding and preference. Oxytocin, as previously mentioned, is a key hormonal messenger during parturition, and sheep have been shown to release large amounts of oxytocin in response to vaginocervical stimulation (6), like that typical during birth. The oxytocin release during birth seems to play a necessary and causal role in not just inducing nurturing behavior in estrogen-primed females (7), like in rats, but also in own-offspring preference and foreign-offspring rejection in sheep ewes, presumably by influencing olfactory memory formation (8). This suggested a key role of oxytocin in maternal social memory and its influence on selective maternal behavior – a role which wasn’t apparent through study of the non-selective parenting of rats.

A natural next question was whether such effects were limited only to female biological parents, or whether in species that show biparental or alloparental care, oxytocin showed similar effects in the non-gestational parents or non-parental caregivers. As neither rats nor sheep show evidence of paternal care or intentional alloparenting, to explore the role of oxytocin in parenting behavior beyond the maternal, researchers turned to yet another model species with a different parenting system and different social system – the prairie vole (*Microtus ochrogaster*). Prairie voles are a socially monogamous rodent species that exhibits biparental care of offspring, and for which the basic social group tends to be a monogamous pair of parents and several litters of their offspring (9, 10). This results in a parenting model that includes both male and female biological parents and alloparents in the form of female offspring from previous litters. With respect to female alloparents, as in rats and sheep, oxytocin seems to be an important driver of parental behavior in virgin female voles: oxytocin receptor density in the nucleus accumbens developed by virgin female prairie voles during the neonatal period is significantly related to the amount of alloparenting behavior that they exhibit toward foreign offspring, both as juveniles and as adults (11). Antagonizing these receptors eliminates alloparental behavior in the voles (11), further indicating a crucial role of oxytocin in alloparents.

However, evidence from prairie voles and other biparental vole species suggests that oxytocin’s role in paternal care is both more subtle and more variable than its role in maternal care. While the overall decrease in testosterone and increase in prolactin are consistent in male parents of multiple species (12–14), fatherhood’s effect on oxytocin (and vice versa) seems to be much more variable and subtle, and although OT receptor binding seems to be upregulated in paternal males, the results are often confounded with cohabitation with the maternal parent in empirical studies of biparental species, meaning that upregulation could simply be due to the presence of a pair-bonded partner (meadow voles, *Microtus pennsylvanicus*: 15; mandarin voles, *Microtus mandarinus*: 16). Further, in prairie voles, fathers may show increased numbers of oxytocin-reactive neurons in the PVN, as well as increased OT-reactive fibers in several other regions (17), but this result is inconsistent (18), even within a species, so it is likely that oxytocin’s effects are both brain-region specific and region-dependent. It might also be that even species-level tendencies toward fatherhood change in the presence of oxytocin. Mouse species vary in their paternal investment based on whether their mating system is monogamous (as in California mice, *Peromyscus californicus*, or mound-building mice, *Mus spicilegus*) or polygamous (for instance, house mice, *Mus musculus*), although most mouse species will show some level of biparental care (19). Even in non-monogamous species, hypothalamic oxytocin neurons regulate levels of paternal care, and in turn, fatherhood strengthens neural connections to these oxytocin neurons (20), suggesting that species-level differences in paternal care are plastic and driven by differences in typical oxytocin expression in those species.

In addition, few studies have characterized if oxytocin changes as a result of fatherhood have behavioral effects, a notable exception being a study in which intranasally administered oxytocin increased tolerance for food transfer to offspring among common marmoset (*Callithrix jacchus*) fathers (21). As another example, administration of intranasal oxytocin in monogamous and biparental California mouse fathers showed lower latency to approach pups following separation than control counterparts (though most other paternal behavior was not significantly affected by oxytocin manipulation: 22). However, direct manipulation of oxytocin in species that show paternal care remains rare, and represents a key area in which non-traditional models can fill an important gap in our knowledge, both in terms of highlighting the role of oxytocin in different social roles as well as determining how oxytocin might play a role in organizing social systems in these models.

The pair-bonding behavior exhibited by some of these biparental model species also provide important evidence for oxytocin’s role in the formation and maintenance of adult relationships in social species. Oxytocin has been shown to play a consistent role in pair-bonding behavior in both female and male prairie voles (although, see our discussion of interactions with other hormones, like arginine-vasopressin, below), and long-term oxytocin administration has effects on affiliative behavior in both members of a titi monkey pair bond (23), with evidence that oxytocin receptor binding in the titi monkey hippocampus was related to affiliative contact between pairmates (24). Further, direct comparison with non-pair-bonding but related species has specified a potential activational role of oxytocin; oxytocin receptor densities differ among different neural regions in the pair-bonding prairie vole and the non-pair-bonding meadow vole (*Microtus pennsylvanicus*), suggesting that oxytocin may activate different pathways as a result of social organization (25).

Even among more gregarious species, in which there are many different types of adult relationships, ranging from breeding pairs to same-sex friendships, oxytocin has been linked to attachment, affiliative bond formation, and maintenance of relationships between adult group members. Further, some of these gregarious species are highly curious, manipulative, and cooperative in a laboratory setting, which provides the opportunity for controlled study of hormonal correlates of behavior and subsequent decision-making. As an example of one such species, capuchin monkeys (*Sapajus apella*) show a reliable increase in oxytocin following grooming and following fur-rubbing, a behavior often done in concert with conspecifics, indicating that these behaviors are serving as a bond maintenance behavior even among non-kin dyads (26, 27). Further, as some captive capuchins are trained to complete cognitive tasks, they represent a unique opportunity to manipulate endogenous oxytocin and study subsequent changes in social attention and behavior (28). Although potentially possible in more traditional model species (for instance, many rhesus macaques are trained to complete cognitive tasks), studying species, like capuchins, with gregarious natures provides the opportunity to study how relationship quality among adults might be related to how oxytocin impacts their social decision-making in a group context (a relationship which is inconsistent and may be related to specific behavioral contexts even within a gregarious species, such as chimpanzees, *Pan troglodytes*: 29, 30). Indeed, inclusion of such species will make it more clear whether oxytocin’s effect is the same across even multiple primate species (both more- and less-similar in social system – for instance, bonobos, *Pan paniscus*: 31), or if species-specific behavioral contexts drive effects.

Thus, our present understanding of oxytocin’s function is not limited to maternal behavior, but through direct examination of its role among varied, non-traditional model species, has widened to include a more general social effect of attachment among individuals of both sexes and among different types of relationships. Interestingly, this understanding of oxytocin’s function is in some ways broader in that oxytocin certainly seems to have social function, but is also far more specific, in that species-specific social contexts and specific receptor densities may greatly affect oxytocin’s impact on observed behavior. Further, oxytocin is by no means the only hormone implicated in social behavior, and the observed outcome of its activity is moderated by a range of other hormones in a given social context that also differ by individual experience, by biological sex, and by response to environmental stimuli. One such example is the likely interplay between oxytocin and arginine-vasopressin (hereafter, vasopressin) in producing paternal offspring care – antagonizing neuropeptide receptors in male prairie voles showed that paternal behavior only declined when both oxytocin and vasopressin receptors were antagonized, rather than one or the other (32), suggesting that both oxytocin and vasopressin play important roles in producing paternal behavior. Complicating matters further, gonadal steroids like testosterone have been shown to have intricate interactions with the oxytocin system to either excite or suppress its activity (as in mice, where testosterone suppresses oxytocin system activity and subsequent paternal sensitization to pups: 33). Thus, in order to gain a full understanding of hormonal correlates of behavior, and how those correlates might influence subsequent behavior, the intentional use of multiple species with a range of social attachment types is useful to determine which functions of oxytocin are present among all species and which of its functions are species- or system-specific.

Indeed, though most mammals have some form of interaction with conspecifics throughout their life, many species do not engage in frequent social interactions, instead remaining largely solitary (for instance, flanged male orangutans, *Pongo* spp., rarely live in groups and most of their social interaction is with unbonded females that live in their home range). Despite this, many of these more solitary mammalian species have oxytocin systems of their own, which begs the question of the role that oxytocin plays in these mammalian species. As an example, domestic housecats (*Felis silvestris*), though they often interact with conspecifics and even appear to form bonds with preferred humans, show little evidence of forming conspecific social groups with attachments. Supporting this, oxytocin levels are far less predictive of social centrality and affiliation in high-density-living cats than are cortisol and testosterone (34), suggesting the possibility that rather than forming attachments with nearby conspecifics, cats are instead merely tolerating feline neighbors. Further, urinary oxytocin increased only when social contact with their typical human caregiver was removed (35). Despite this, exogenous oxytocin alters social attention in male cats toward humans (though the effect was sex-specific: Hattori et al. (36)). Thus, it might be that oxytocin responses differ along the spectrum of social relationships, and oxytocin-mediated behavior varies as a result of typical oxytocin response as well as having attachment-specific responses. To fully understand this, incorporating more solitary mammals of multiple taxa will provide important information for comparison.

Although our review is focused on the exploration of non-traditional mammalian models (summarized in **Table 1**), we highlight the utility of including non-mammalian and even invertebrate models in our understanding. Extending our understanding of oxytocin (or more specifically, its analogues) beyond vertebrate species is a relatively new avenue of research that highlights how the addition of even more non-traditional models for oxytocin action can help us to understand both the generalized social effects of oxytocin and the species-specific behavioral effects. Oxytocin-related peptides (as described in 72) are present even in invertebrate species (for instance, several species of social ants, 73), and there is evidence for a role of the oxytocin-related peptide inotocin in social foraging organization of raider ants (*Ooceraea biroi;* 74), suggesting that oxytocin and similar neuropeptides represent an evolutionarily ancient system involved in reproduction and social signaling among animals. However, the notable absence of oxytocin-like signaling in other highly social insect models, such as the honeybee (*Apis mellifera*: 72, 75), provides an intriguing opportunity to explore what other mechanisms or hormones may be filling this role, how social behavior might have evolved even in the absence of oxytocin, and how oxytocin might have changed this evolutionary trajectory. Notably, it is only through intentional inclusion of these non-traditional animal models that we will be able to fully explore the evolution of these complex social behaviors throughout the full animal lineage.

**Table 1:** Summary table of representative oxytocin literature across mammalian species by year, including mating system and parental system for comparison.

Authors	Year	Species	Mating System	Social System	Parental System	Sex	Total Sample Size	Behavior Studied or Outcome Measure	Manipulated OT? How?	Measured OT? How?	Summary of Effects of OT	Notes
Pedersen & Prange (2)	1979	Rats	Promiscuous	Multi-male-multi-female	Uniparental maternal	Females	35	Maternal behavior	Manipulated, exogenous intracerebroventricular OT		↑ OT = ↑ maternal response in virgin rats	
Bolwerk & Swanson (3)	1984	Rats	Promiscuous	Multi-male-multi-female	Uniparental maternal	Females	36	Parental care	Manipulated, exogenous introcerebroventricular OT		No effect of OT	Possible strain differences, or interaction with estrogen
Kendrick et al. (6)	1986	Domestic sheep	Promiscuous	Multi-male-multi-female	Uniparental maternal (selective)	Females	21	Birth and post-partum nursing		Measured, cerebrospinal fluid (CSF) OT	↑ OT associated with labor, parturition, and nursing; ↑ OT also associated with vaginocervical stimulation in normally-cycling ewes	
Kendrick et al. (7)	1987	Domestic sheep	Promiscuous	Multi-male-multi-female	Uniparental maternal (selective)	Females	7	Maternal behavior in ovarectomized and estrogen-treated ewes	Manipulated, exogenous intracerebroventricular OT		↑ OT = ↑ sniffing, licking, approaching, allowing suckling, ↓ aggression and withdrawal	Vaginocervical stimulation produced similar effects as exogenous OT
van Leengoed et al. (4)	1987	Rats	Promiscuous	Multi-male-multi-female	Uniparental maternal	Females	14	Maternal behavior	Manipulated, exogenous intracerebroventricular OT		↓ OT = ↓ maternal behavior, ↑ latency to group pups	
Insel & Shapiro (25)	1992	Prairie voles	Socially monogamous	Pair-bonded	Biparental	Both	3 per sex for each brain region	OT receptor binding and parental behavior		Measured, OT receptor binding	Prairie voles showed higher OT receptor binding in the prelimbic cortex, the bed nucleus of the stria terminalis, the nucleus accumbens, midline nuclei of the thalamus, and lateral amygdala; montane voles showed little binding in these areas	Direct comparison between monogamous and polygynous vole species (see entry for montane voles as well)
Insel & Shapiro (25)	1992	Montane voles	Promiscuous	Multi-male-multi-female	Uniparental maternal	Both	3 per sex for each brain region	OT receptor binding and parental behavior		Measured, OT receptor binding	Prairie voles showed higher OT receptor binding in the prelimbic cortex, the bed nucleus of the stria terminalis, the nucleus accumbens, midline nuclei of the thalamus, and lateral amygdala; montane voles showed little binding in these areas	Direct comparison between monogamous and polygynous vole species (see entry for prairie voles as well)
Lévy et al. (8)	1995	Domestic sheep	Promiscuous	Multi-male-multi-female	Uniparental maternal (selective)	Females	28	Maternal experience and behavior	Manipulated, exogenous intracerebroventricular OT	Measured, olfactory bulb circulating OT	↑ endogenous OT associated with multiparous females at birth as compared to primiparous females; ↑ intracerebroventricular OT = ↑ acetylcholine in multiparous females, ↑ noradrenaline in multiparous females, and ↑ GABA in both	
Parker et al. (15)	2001	Meadow voles	Promiscuous	Multi-male-multi-female	Uniparental maternal	Males	34	Sexual and paternal experience and behavior		Measured, OT receptor density	↑ OT receptor density in the anterior olfactory nucleus, bed nucleus of the stria terminalis, lateral septum, and lateral amygdala associated with sexual/paternal experience	Study also associated AVP receptor binding in several of these regions
Bales et al. (32)	2024	Prairie voles	Socially monogamous	Pair-bonded	Biparental	Males	80	Parental care	Manipulated, OT receptor antagonist (OTA)		No effect of OTA alone	When paired with AVP antagonist, ↓ OT + ↓AVP = ↓ huddling, ↓ parental behavior
Kosfield et al. (37)	2005	Humans	Varies based on culture	Fission-fusion	Biparental/Cooperative	Males	194	Trust	Manipulated, exogenous intranasal OT		↑ OT = ↑ responses indicating trust in a monetary allocator in an allocation game	↑ trust responses independent of risk-taking tendencies
Takayanagi et al. (5)	2005	Laboratory mouse	Promiscuous	Depends on commensality	Uniparental maternal, with some biparental care varying	Both	unknown	Maternal and social behavior	Manipulated, OT receptor knockout strain		↓ OT = ↓ social behavior, ↓ maternal behavior, ↓ social discrimination, no effect on parturition	
Olazábal & Young (11)	2006	Prairie voles	Socially monogamous	Pair-bonded	Biparental	Females	9	Juvenile alloparental behavior		Measured, OT receptor density	↑ OT receptor density in nucleus accumbens and caudate putamen and ↓ OT receptor density in lateral septum in prairie voles (which readily exhibit alloparental care) as compared to rats, mice, and meadow voles (which either show alloparental care only after several days or never show alloparental care)	Compare to entries for meadow voles, laboratory mice, rats
Olazábal & Young (11)	2006	Meadow voles	Promiscuous	Multi-male-multi-female	Uniparental maternal	Females	6	Juvenile alloparental behavior		Measured, OT receptor density	↓ OT receptor density in nucleus accumbens and caudate putamen and ↑ OT receptor density in lateral septum in meadow voles (which rarely or never show alloparental care) as compared to prairie voles (which readily exhibit alloparental care)	Compare to entries for prairie voles, laboratory mice, rats
Olazábal & Young (11)	2006	Laboratory mouse	Promiscuous	Depends on commensality	Uniparental maternal, with some biparental care varying	Females	7	Juvenile alloparental behavior		Measured, OT receptor density	↓ OT receptor density in nucleus accumbens and caudate putamen and ↑ OT receptor density in lateral septum in laboratory mice (which rarely or never show alloparental care) as compared to prairie voles (which readily exhibit alloparental care)	Compare to entries for prairie voles,meadow voles, rats
Olazábal & Young (11)	2006	Rats	Promiscuous	Multi-male-multi-female	Uniparental maternal	Females	8	Juvenile alloparental behavior		Measured, OT receptor density	↓ OT receptor density in nucleus accumbens and caudate putamen and ↑ OT receptor density in lateral septum in rats (which rarely or never show alloparental care) as compared to prairie voles (which readily exhibit alloparental care), though rats OT receptor density tended to be intermediate compared to meadow voles or laboratory mice	Compare to entries for prairie voles,meadow voles, laboratory mice
Ring et al. (38)	2006	Laboratory mouse	Promiscuous	Depends on commensality	Uniparental maternal, with some biparental care varying	Males	unknown	Anxiety responses	Manipulated, intracerebroventricular OT agonist and antagonist		↑ OT = ↑ punished crossings in four-plate test, ↑ time spent in open quadrants of elevated zero maze	
Bales et al. (39)	2007	Prairie voles	Socially monogamous	Pair-bonded	Biparental	Females	60	Parental care	Manipulated, exogenous injection OT within 24 hours at birth		Dose-dependent effect such that certain doses resulted in ↑ OT = ↑ pup retrieval, ↓latency to retrieve pups	
Zak et al. (40)	2007	Humans	Varies based on culture	Fission-fusion	Biparental/Cooperative	Males	68	Generosity and donation behavior	Manipulated, exogenous intranasal OT		↑ OT = ↑ generosity in the ultimatum game, no effect on donation in the dictator game	
Baumgartner et al. (41)	2008	Humans	Varies based on culture	Fission-fusion	Biparental/Cooperative	Males	49	Trust	Manipulated, exogenous intranasal OT		↑ OT = ↓ fear of social betrayal following betrayal in dyadic game; ↓ latency to make trusting decision; ↓ activation of amygdala and uncleus accumbens following betrayal	
Morhenn et al. (42)	2008	Humans	Varies based on culture	Fission-fusion	Biparental/Cooperative	Both	96	Physical touch and monetary sacrifice		Measured, plasma and/or serum OT	↑ OT associated with receiving physical touch in the form of massage; ↑ OT as a result of massage associated with increased donations to strangers in a trust game	
Snowdon et al. (43)	2010	Cotton-top tamarins	Socially monogamous	Multi-male-multi-female	Cooperative	Both	28	Affiliative behavior		Measured, urinary OT	OT synchronized between mates within pair, but no sex difference; ↑ OT associated with ↑ sexual behavior, ↑ grooming, ↑ huddling	
Song et al. (16)	2010	Mandarin voles	Socially monogamous	Pair-bonded	Biparental	Males	35	Alloparental behavior		Measured, OT immunoreactivity	↑ OT associated with paternal experience (which was also positively related to alloparental behavior)	
Saito & Nakamura (21)	2011	Common marmosets	Socially monogamous	Pair-bonded	Cooperative	Males	6	Paternal behavior	Manipulated, exogenous intranasal OT		↑ OT = ↑ tolerance of food transfer from father to offspring	
Chang et al. (44)	2012	Rhesus macaques	Promiscuous	Multi-male-multi-female	Uniparental maternal	Males	2	Reward donation	Manipulated, exogenous intranasal OT		↑ OT = ↑ attention to recipient,↑ donation to recipient, ↑ self-reward	
De Dreu et al. (45)	2012	Humans	Varies based on culture	Fission-fusion	Biparental	Males	72	Response to intergroup conflict	Manipulated, exogenous intranasal OT		↑ OT = ↑ alliances with high-threat individuals, ↑ protection of in-group	
Moscovice & Ziegler (46)	2012	Bonobos	Promiscuous	Fission-fusion	Uniparental maternal	Females	13	Estrous status and sexual consortship		Measured, urinary OT	↑ OT associated with periovulatory period of estrous cycle; no associated between OT and consortship status, but ↑ OT associated with closer proximity in consorting females	Consortship defined as a short-term, exclusive mating relationship with a male conspecific
Crockford et al. (47)	2013	Chimpanzees	Promiscuous	Fission-fusion	Uniparental maternal	Both	33	Social bonding		Measured, urinary OT	↑ OT associated with grooming in kin dyads and non-kin bonded dyads, but not in non-bonded dyads	
Ebitz et al. (48)	2013	Rhesus macaques	Promiscuous	Multi-male-multi-female	Uniparental maternal	Males	7	Social vigilance	Manipulated, exogenous intranasal OT		↑ OT = ↓ attention to social stimuli, ↑ response time to social stimuli	
Okabe et al. (33)	2013	Laboratory mouse	Promiscuous	Depends on commensality	Uniparental maternal, with some biparental care varying	Both	76			Measured, OT immunoreactivity	↓ Testosterone = ↑ OT immunoreactive neurons in both species (no effect observed for estrogen)	Sample size across multiple condition groups
Striepens et al. (49)	2013	Humans	Varies based on culture	Fission-fusion	Biparental/Cooperative	Males	15	Physiological measurement of OT following exogenous administration	Manipulated, exogenous intranasal OT	Measured, plasma and urinary OT	↑ exogenous OT = ↑ endogenous OT (both plasma and CSF), but pharmokinetic timescale differs, such that plasma OT rises and peaks much more quickly	
Kenkel et al. (17)	2014	Prairie voles	Socially monogamous	Pair-bonded	Biparental	Males	28	Paternal behavior		Measured, OT receptor density and immunoreactivity	Fathers showed ↑ OT-immunoreactive neurons in the paraventricular nucleus of the hypothalamus than virgins, as well as ↑ fiber density in the nucleus accumbens and nucleus tractus solitarius; however, fathers showed ↓ OT neurons in the bed nucleus of the stria terminalis	
Rilling et al. (50)	2014	Humans	Varies based on culture	Fission-fusion	Biparental/Cooperative	Females	87	Reciprocal altruism and cooperation	Manipulated, intranasal OT		↑ OT = women treated computer partners more similarly to when they were playing against a human; ↑ OT also either did not affect or resulted in ↓ neural activity in the striatum, basal forebrain, insula, amygdala, and hippocampus (all regions which showed increased activity in men)	Male results came from previous data collection of same paradigm
Romero et al. (51)	2014	Domestic dogs	Promiscuous	Multi-male-multi-female	Uniparental maternal	Both	16	Social bonding and affiliative behavior	Manipulated, exogenous intranasal OT	Measured, plasma and urinary OT	↑ exogenous OT = ↑ plasma OT,↑ urinary OT, ↑ affiliation with owners, ↑ affiliation with conspecifics, ↓ high-frequency heart rate variability	
Weinstein et al. (52)	2014	Rhesus macaques	Promiscuous	Multi-male-multi-female	Uniparental maternal	Both	54	Friendships and social affiliation		Measured, plasma OT	↑ OT in adulthood had a U-shaped relationship with number of reciprocal and play friendships during juvenile period in females (no effect in males)	
Wittig et al. (53)	2014	Chimpanzees	Promiscuous	Fission-fusion	Uniparental maternal	Both	26	Food sharing		Measured, urinary OT	↑ OT associated with ↑ food sharing during feeding events	
Brosnan et al. (54)	2015	Tufted capuchin monkeys	Promiscuous	Multi-male-multi-female	Uniparental maternal	Both	8	Food sharing and social distance	Manipulated, exogenous intranasal OT		↑ OT = ↓ passive food transfer, ↑ social distance	
Carter & Wilkinson (55)	2015	Vampire bats	Promiscuous	One-male-multi-female	Uniparental maternal	Both	5	Food sharing and allogrooming	Manipulated, exogenous intranasal OT		↑ OT = ↑ amount of food donation to conspecific (but no effect on food sharing occurrence), ↑ duration of allogrooming (but no effect on allogrooming occurrence)	
Cavanaugh et al. (56)	2015	Common marmosets	Socially monogamous	Pair-bonded	Cooperative	Both	12	Affiliation with pair mate	Manipulated, intranasal OT agonist and oral OT antagonist		↑ OT = ↑ proximity in both sexes, ↑ grooming in females	Both Pro^8^ OT and Leu^8^ OT administered (endogenous OT is Pro^8^ in this species)
Feng et al. (57)	2015	Humans	Varies based on culture	Fission-fusion	Biparental	Both	304	Neural responses to social cooperation	Manipulated, exogenous intranasal OT		For males: ↑ OT = ↑ activation in caudate, right frontal pole, and left medial part of superior medial frontal cortex; for females, ↑ OT = ↓ activation in these regions	Administration of intranasal AVP also explored
Burkett et al. (58)	2016	Prairie voles	Socially monogamous	Pair-bonded	Biparental	Both	28	Consolation behavior	Manipulated, OT receptor antagonist (OTA)		↓ OT = ↓ allogrooming, ↓ partner-directed behavior	Part of larger examination of consolation, which also included female prairie voles and comparison with meadow voles; we report only oxytocin-related findings
Finkenwirth et al. (59)	2016	Common marmosets	Socially monogamous	Pair-bonded	Cooperative	Both	26	Infant care and motivation		Measured, urinary OT	↑ OT associated with early post-partum period; ↑ OT associated with breeder females as compared to helpers or males	
Proctor et al. (29)	2016	Chimpanzees	Promiscuous	Fission-fusion	Uniparental maternal	Females	8	Social behavior	Manipulated, exogenous intranasal OT		No effect of OT on proximity, grooming, or general activity level	
Samuni et al. (60)	2017	Chimpanzees	Promiscuous	Fission-fusion	Uniparental maternal	Both	20	Intergroup conflict		Measured, urinary OT	↑ OT associated with immediate pre-conflict and mid-conflict period, ↑ ingroup cohesion during conflict	
Benítez & Sosnowski et al. (26)	2018	Tufted capuchin monkeys	Promiscuous	Multi-male-multi-female	Uniparental maternal	Both	18	Affiliation	Manipulated, exogenous intranasal OT	Measured, urinary OT	↑ intranasal OT = ↑ urinary OT; ↑ affiliative behavior (grooming, fur-rubbing) = ↑ urinary OT	Pro^8^ OT
Guoynes et al. (61)	2018	Prairie voles	Socially monogamous	Pair-bonded	Biparental	Both	173	Neural responses to chronic exogenous OT	Manipulated, exogenous intranasal OT		For females: ↑ OT = ↑ OT receptor binding in the nucleus accumbens; for males, no effect of OT	Also explored chronic OT effect on AVP receptors (no effect for either)
Rilling et al. (62)	2018	Humans	Varies based on culture	Fission-fusion	Biparental/Cooperative	Both	304	Social interaction	Manipulated, intranasal OT		↑ OT = ↑ functional connectivity in response to positive social interactions in men, ↓ functional connectivity in response to negative social interactions in women	
Preis et al. (63)	2018	Chimpanzees	Promiscuous	Fission-fusion	Uniparental maternal	Males	10	Post-conflict reconciliation		Measured, urinary OT	↑ OT associated with post-conflict reconciliation and affiliation, as compared to non-reconciled aggression	
Moscovice et al. (64)	2019	Bonobos	Promiscuous	Fission-fusion	Uniparental maternal	Females	13	Sexual behavior		Measured, urinary OT	↑ OT associated with same-sex sexual behavior as compared to baseline or copulation with males	
Samuni et al. (65)	2019	Chimpanzees	Promiscuous	Fission-fusion	Uniparental maternal	Both	20	Intergroup conflict		Measured, urinary OT	No association between OT and increased levels of outgroup hostility and contact during intergroup conflict (although conflict was generally associated with ↑ OT)	
Smith et al. (66)	2019	Tufted capuchin monkeys	Promiscuous	Multi-male-multi-female	Uniparental maternal	Both	12	Social decision-making	Manipulated, exogenous intranasal OT		No effect of OT on strategy formation or maintenance in the Assurance, Hawk-Dove, or Prisoner’s Dilemma games	
Baxter et al. (24)	2020	Coppery titi monkeys	Socially monogamous	Pair-bonded	Biparental	Both	10	Parental care and pair bonding		Measured, oxytocin receptor (OTR) binding	Parenting associated with ↑ OTR binding in hippocampus; ↑ OTR binding associated with ↑ contact and proximity with bonded partner	
Declerck et al. (67)	2020	Humans	Varies based on culture	Fission-fusion	Biparental	Males	677	Trust	Manipulated, exogenous intranasal OT		No effect of OT on trust in minimal social contact situations	
Martins et al. (68)	2020	Humans	Varies based on culture	Fission-fusion	Biparental/Cooperative	Males	17	Neural responses to exogenous OT	Manipulated, exogenous intranasal and intravenous OT		Both methods of ↑ OT = ↓ amygdala activity	Also compared multiple methods of intranasal administration, with key differences in the pattern of neural activity that suggest that nebulizer administration may be a more effective tool
Rincon et al. (69)	2020	Barbary macaques	Promiscuous	Multi-male-multi-female	Uniparental maternal	Males	14	Affiliation		Measured, urinary OT	↑ OT associated with ↑ grooming interactions and triadic male-infant-male interactions with non-bond partners (but not after interactions with bond partners)	
Arias-del Razo et al. (23)	2022	Coppery titi monkeys	Socially monogamous	Pair-bonded	Biparental	Both	29	Pair bonding	Manipulated, exogenous intranasal OT		↑ OT = ↑ affiliation, ↑ partner preference (males), ↑ aggression toward strangers (males)	
Brooks et al. (31)	2022	Bonobos	Promiscuous	Fission-fusion	Uniparental maternal	Both	5	Social gaze	Manipulated, exogenous intranasal OT		↑ OT = ↑ eye contact	OT exacerbated existing species-level tendencies (see entry for chimpanzees)
Brooks et al. (31)	2022	Chimpanzees	Promiscuous	Fission-fusion	Uniparental maternal	Both	6	Social gaze	Manipulated, exogenous intranasal OT		↑ OT = ↓ eye contact	OT exacerbated existing species-level tendencies (see entry for bonobos)
Inada et al. (20)	2022	Laboratory mouse	Promiscuous	Depends on commensality	Uniparental maternal, with some biparental care varying based on species	Males	unknown	Neural plasticity and paternal behavior	Manipulated, OT conditional knockout		OT neurons induce paternal behavior even in virgin males; synaptic plasticity in OT neurons in fathers results in observed behavioral plasticity	
Kou et al. (70)	2022	Humans	Varies based on culture	Fission-fusion	Biparental/Cooperative	Males	147	Anxiety and neural activity	Manipulated, chronically administered exogenous intranasal OT		Low-frequency of ↑ OT = ↓ reactivity to threatening stimuli in amygdala - insula - prefrontal circuits in self-reported high anxious subjects	
Sosnowski et al. (28)	2022	Tufted capuchin monkeys	Promiscuous	Multi-male-multi-female	Uniparental maternal	Both	5	Social gaze	Manipulated, exogenous intranasal OT and induced release of endogenous OT		↑ endogenous OT = ↑ frequency of looking toward the eye region, ↓ duration of looking at eye region; ↑ exogenous OT = ↓ duration of looking at eye region	
Guoynes et al. (22)	2023	California mice	Socially monogamous	Pair-bonded	Biparental	Both	99	Social preferences responses to exogenous OT	Manipulated, exogenous intranasal OT		↑ OT = ↑ peri-adolescent male social preference for parents over peers (no effect for females)	No effect in tests intended to study the effect of OT on stress or anxiety (elevated plus maze and novel object test)
Sosnowski et al. (27)	2023	Tufted capuchin monkeys	Promiscuous	Multi-male-multi-female	Uniparental maternal	Both	9	Fur-rubbing/anointing		Measured, urinary OT	↑ OT associated with fur-rubbing with an onion, regardless of physical or visual contact with conspecifics	
Witczak et al. (71)	2024	Coppery titi monkeys	Socially monogamous	Pair-bonded	Biparental	Both	40	Father-daughter relationships and social behavior	Manipulated, exogenous intranasal OT		Greater affiliation with father interacted with ↑ OT in dose-dependent manner in producing responses to parent preference test (as compared to stranger presence)	

↓, refers to a decrease in expression or activity; ↑, refers to an increase in expression or activity.

## 3 Oxytocin’s variable effects on social behavior

Beyond social systems, research has focused how oxytocin may be influencing other social behaviors. The rationale is that if oxytocin was influencing pair bonding and mother-offspring bonds, it might also play a role in other important social relationships. Although this need not necessarily be the case, given some of the unique aspects of these bonds, especially between mother and offspring, research has suggested that oxytocin does, in some cases, influence them. Much of the earlier work was done with humans. The typical study gave humans an exogenous dose of oxytocin, through an inhaler, and measured how oxytocin, versus a placebo, influenced prosocial behaviors. As predicted, various studies found that humans were more likely to show behaviors such as giving money in economic games (40, 42) and increasing trust (37, 41), and possibly even showed changes in neural circuitry after inhaled oxytocin (62). Touch, a stimulus that also elicits endogenous oxytocin release, also increases affiliative behaviors (76). After this initial push, however, later studies suggested that these results were overly simplistic (77), with other studies finding more variable effects, in particular that oxytocin also increased aggression towards outgroups (45). In addition, few human studies have used female participants, yet those that do suggest that this is an important oversight, with females often showing very different behavior than males in the same context (50, 57, 78).

Work in non-human species lagged behind, in part because of the challenge of measuring or administering oxytocin in these species. The former requires an invasive spinal tap to measure central oxytocin, or is reliant on measuring peripheral measures (i.e., urine) and making assumptions about what changes in peripheral oxytocin tell us about central oxytocin levels. The latter requires either very invasive procedures (i.e., intercranial injections) or significant training for the animal to remain still for inhaled administration (which, data suggest, does result in oxytocin crossing the blood brain barrier; 49, 68, 79, 80). There is a trade-off between these, as more invasive procedures are typically more accurate, but may not be possible, for ethical or practical reasons, in species that are group housed in social contexts or for individuals tested with pairs. However, these results are essential to determine the degree to which the results found in humans are general primate processes, or a result of distinct human selective pressures.

Studies exploring how different behaviors influence the natural release of endogenous oxytocin have generally supported the view that oxytocin is linked to prosocial behaviors, with behaviors such as grooming (47, 53, 63, 69), cooperating (i.e., during intergroup conflict; (60, 65), and sexual behaviors (43, 46, 64) increasing peripheral oxytocin measures. These studies have largely focused on chimpanzees in the field, capuchin monkeys, and marmosets, all of which are well documented to show cooperative behaviors in field and lab settings (chimpanzees: 81–83; capuchins: 84; marmosets: 85, 86), suggesting that it would be good to study other species that are not expected to be as cooperative; in other contexts, this approach has found unexpected subtleties in the relationship between demographics and behavior (87, 88). Indeed, there are nuances to oxytocin’s effect in these social contexts. For instance, chimpanzees’ grooming following oxytocin increases more when the interaction is between kin or individuals who share strong affiliative bonds as opposed to unbonded individuals (47). We therefore anticipate that relationship quality might be an important factor, as it also can influence cooperative behavior in and of itself (for instance, in marmoset infant care: 89), so relationship quality likely interacts with oxytocin when considering cooperative acts (again, in marmoset infant care: 59). However, there often are not sufficient interactions between non-bonded individuals to determine whether there is an effect of oxytocin (26), so assessing the full range of relationship quality may be challenging when considering this interaction.

Of course, measuring how behavior changes oxytocin is only half of the story; to see the interplay between oxytocin and behavior, we also need to know how manipulating oxytocin influences behavioral outcomes. Despite results from mammalian species suggesting that exogenous oxytocin might influence behavior in those species (dogs, *Canis familiaris*: 51; vampire bats, *Desmodus rotundus*: 55; prairie voles: 58), the results of such studies have been somewhat inconsistent in primates (although some other evidence shows similar behaviors in non-primate mammals). Although there are some results that suggest that inhaling oxytocin increases subsequent prosocial behaviors in primates, including donating to one’s partner (44), grooming (31), consolation (58), increased social bonding (51), or other changes in behavior (21, 48), a large number of studies have found no effect. For instance, intranasal oxytocin did not increase any of several prosocial behaviors in marmosets, other than the long term mate (56), nor did it increase coordination on an economic game in capuchins (66) or chimpanzees (90). Results from grooming are more complex. Providing inhaled oxytocin did not increase grooming in chimpanzees (29) and only one bonobo showed an increase in another study (31). Adding to the confusion, a study of capuchin subjects showed a behavioral pattern following intranasal oxytocin in which grooming decreased, then increased, before falling back to baseline levels (26), suggesting that there may be dynamic effects across time and that studies that measure a single timepoint may miss this and suggest “negative” or contradictory findings.

It is not entirely clear why there is so much inconsistency. One possibility is that oxytocin is influencing core behavioral mechanisms in ways that result in different behaviors in different systems; for instance, considering eye gaze in primates, giving inhaled oxytocin increases bonobos’ gaze towards eyes, but chimpanzees’ and capuchins’ gaze towards the periphery of the face. However, these inconsistent results can be explained by a single underlying mechanism, which is oxytocin increasing the species’ natural propensities. Indeed bonobos spend more time than the other species looking at eye regions under baseline conditions (28, 91, 92). It is also possible that different dosages of oxytocin lead to different behaviors; there is some evidence that oxytocin’s effects may follow an inverted-U based on dosing (28, 39, 61). This could affect both instantiation of behavior and, possibly, timing of effects (for instance, the grooming effects discussed above). Finally, it is not clear that inhaled oxytocin influences behavior in the same way as naturally released oxytocin, and recent work with capuchin monkeys suggests that behavior after induced natural release (i.e. through fur rubbing) may be different than behavior following exogenous inhaled oxytocin (26, 28), although dose-matched work has not been done in comparing externally-administered and internally-released oxytocin. Future work with different species will be essential to determine if exogenous oxytocin reacts in the same way as endogenously released oxytocin.

## 4 Benefits and challenges of different species and taxa comparisons

The use of different species with differing social systems to understand the role of social biomarkers provides a unique opportunity to fully understand the range of effects of a given hormone, as well as practical benefits for endocrinological study. First, we note that it is not unreasonable to explore hormones in traditional models, where we are beginning to understand their effects. Indeed, from a logistical standpoint, many traditional mammalian models (for instance, rhesus macaques, *Macaca mulatta*, or the laboratory rat, *Rattus norvegicus*) have been chosen not just for physiological similarity to humans, but also due to the ease of captive husbandry. Indeed, due to unique social needs or unusual species-typical behaviors, non-traditional mammalian models may have specific or challenging husbandry or enrichment needs to meet. However, a sole focus on traditional model species at the expense of social systems that are less easily maintained in a captive setting leads to a model of endocrinological function that does not account for the subtle function of hormones to produce species-specific behavior or to drive complex social affiliation among different types of adult relationships. Different species exhibit differing behavioral and cognitive abilities (some of which may be evolutionarily linked to differing social systems), and these differences may provide key insights into the function of hormones in behavior even within the same taxa. Recent initiatives have begun to address some of the logistical challenges presented by social systems in which large numbers are difficult to maintain. In part, this has been done through collaborations between laboratory and field researchers, but care needs to be taken to ensure scientific rigor across study sites. Indeed, this goal is a focus of the new movement towards cross-lab collaborative science, which in its most extreme manifestation takes the form of Big Team Science approaches such as ManyPrimates (93), or ManyDogs (94).

Taking primates as an example, the most typical model primate species is the rhesus macaque, which in the wild exhibits a deeply hierarchical social system based on matrilineal relationships within a larger group (95); thus, dominance is highly structured and enforced among females through within-kin support in conflicts and within-matrilineal affiliation, and males must leave their natal group to form new alliances and compete for reproductive access (96). Therefore, the most important adult relationships for a female rhesus macaque tend to be her mother and her sisters, and rhesus macaque females have few adult “friendships” with other females or long-lasting bonds with the alpha male in their group that extend beyond the estrus phase of the reproductive cycle (96). Of course, oxytocin can be explored in the context of rhesus macaque relationships, and macaques have been an important model for activational roles of oxytocin in captive settings – previous literature has focused on the role of oxytocin in social motivation (44) and social vigilance (48). Additionally, there is evidence from group-living macaques linking the number of early same-sex friendships to oxytocin in female rhesus macaques (52). However, due to their highly kin-based hierarchy of social groups and the lack of long-term bonding between the alpha male and most of the females in the group, rhesus macaques make a poor model for many of the adult relationships that humans develop and maintain – for instance, between a long-term sexual partner or between closely bonded same-sex non-kin relationships in both sexes.

On the opposite end of the spectrum as the traditional model, titi monkeys have an extremely narrow social ecology, in which the basic unit of sociality is comprised of the two pair-bonded breeding adults and any offspring that they might have at a given time (97, 98). While there is some behavioral flexibility in this group composition based on the relative ease of offspring dispersal (99), titi monkey groups tend to be relatively small and based largely on kin-relationships to the breeding pair. Thus, for titi monkeys, the most important social bond is between the two breeding adults, and notably, titi monkey males have little reproductive competition once a pairbond has been formed and relatively little social stress. The need for separate housing for titi monkey groups due to their specific social needs (and their distinct rejection of monkeys outside of their small social groups, especially as the bond is still forming: 100) can make titi monkeys a more challenging species to maintain in a laboratory setting, especially given the quality of care necessary to maintain species-typical social behavior. However, titi monkeys are one of the only primate species in which we can study the role of oxytocin in pair bond formation, which make them an important endocrinological model against which to explore oxytocin’s role in the formation of romantic partnership. Indeed, titi monkeys are a key species in which there has been a demonstrated interaction between pair bond status and oxytocin expression (23, 24), suggesting that there are important similarities with pair-bonded non-primate species, like prairie voles; however, differences in oxytocin receptor expression in the brain between primate and rodent models (101) indicate that there are subtle differences in mechanism even within pair-bonded species that need to be explored at species and individual levels.

Neither rhesus macaques (which form relationships based on a strict matrilineal hierarchy) nor titi monkeys (which live in social units consisting of one socially monogamous breeding pair) have multiple types of adult relationships among both sexes. Representing a moderate of the two extremes, capuchin monkeys live in larger, multi-male, multi-female groups, often with several kinship lines (102), resulting in groups with multiple different types of relationships between same-sex and opposite-sex individuals (103–106). Thus, capuchins represent a model species in which we might be able to tease apart subtle roles of oxytocin in the formation of multiple types of relationship (breeding, non-kin “friendships”, and kin relationships). Further, as capuchins frequently show evidence of cooperative behavior (107, 108) or coordinating their behavior with a partner’s choices (84, 109) in experimental tasks, they represent an excellent model species in which to explore how oxytocin might influence the likelihood of cooperation among non-kin individuals. Although previous literature did not find an effect of exogenous oxytocin on food-sharing following intranasal administration in capuchins (54), this study administered the more common form of conserved mammalian oxytocin (Leu^8^ oxytocin) rather than the form that naturally occurs in many *Platyrrhine* monkey species, including capuchins (110; see below). This will be important to test further. Rhesus macaques are not as good of a model for cooperation because they show less evidence of non-kin cooperation (111), and while titis almost certainly coordinate their actions with their pairmate in the course of bond formation and offspring rearing (for instance, during the species-typical duetting between the members of the breeding pair: 112, 113), there has been no empirical testing of cooperative action in this species. Thus, specific testing of cooperative behavior in titi monkeys could provide another avenue to better understand the function of oxytocin in coordinating behavior within a pair, and would provide an important comparison to both the traditional, less-cooperative rhesus macaques and the highly cooperative capuchins.

From a measurement and endocrinological standpoint, there are also benefits to using non-traditional models. Different species may express different biomarkers of physiological systems – for instance, while salivary alpha amylase is an often used biomarker of the sympathetic stress response in humans (114), not all primates express it, probably due to differences in feeding ecology (115); thus, if we want to explore sympathetic responses to stress and how those responses might interact with oxytocin, it would be inappropriate to choose a species which does not express the gene. Further, even when traditional model species express a given hormone, there may be species-level differences in the structure of that gene. Oxytocin differs in key ways among the primate lineage – namely, many (but not all) *Platyrrhine* monkeys express a form of oxytocin with an amino acid switch (known as Pro^8^ oxytocin: 110). While this form of oxytocin seems to have similar effects to the traditional, conserved form (Leu^8^ oxytocin), this difference is points to a divergence in the primate lineage that begs the question of why only some *Platyrrhine* monkeys express the conserved form while others express Pro^8^ oxytocin. Further, if there are subtle binding differences that may lead to downstream behavioral changes (116, 117), it is important to study both forms of oxytocin in *Platyrrhine* species to assess which changes are due to the different form, and which changes are due to other species-level differences. Of course, the differences between these two oxytocin forms can only be studied in these non-traditional *Playtyrrhine* models, where they occur (for instance, comparing titi monkeys [Leu^8^] to capuchin monkeys [Pro^8^]), but understanding how this molecular change does (or does not) affect downstream behaviors may be informative as to how and when oxytocin influences behavior.

Despite the benefits of studying non-traditional model species, we note that the intentional inclusion of these species may also pose specific challenges or considerations. In terms of measurement, to ensure rigor and precision, each hormonal assay must be validated for each species and each sample type that is collected, which can represent a significant time-effort and financial barrier to adding non-traditional species. From a theoretical standpoint, direct comparison of the hormonal effect of behaviors across species may be difficult, as behaviors may represent subtly different things in different species, and the interpretation of these behaviors may be difficult – as one example, adult rhesus macaques associate prolonged direct eye contact as a threat (118), while capuchins and titi monkeys are more tolerant of eye contact and eye contact may even be a key feedback mechanism of affiliative behavior, at least in capuchins (119, 120). This suggests that social structure might be an explanation for differences in eye contact tolerance among the primates (121), and that any hormonal correlates of eye contact need to be interpreted differently among these species.

In addition, different species may have different behavioral repertoires, meaning that there is no direct behavioral comparison, or behaviors may instantiate differently, making it difficult to compare different social behaviors across species. For instance, some white-faced capuchins (*Cebus capuchinus*) display a number of unusual social behaviors that have been described as “trust games” – behaviors performed between two affiliated individuals within a group that seem to serve only to strengthen the existing affiliative relationship; examples of these “games” include a behavior in which one individual places and leaves their fingers within another’s mouth and a behavior in which individuals will gently but firmly take an object back-and-forth from each other’s mouths (122). Of course, such a behavior would be exceptionally risky when performed with a stranger, but with an affiliated individual, these behaviors can serve as reinforcement that neither will react aggressively toward the other - essentially a test of the social bond. These “trust games” are unusual among non-humans, and represent a way to explore how such a test of the social bond might be related to changes in oxytocin across the bond. If oxytocin is generally related to maintaining a social bond, we predict that these games would be associated with increased oxytocin following a bout, suggesting that oxytocin is involved in trust as a key feature of positive relationships not just in humans (37, 41, 67; though also see 123), but in primates and perhaps other taxa generally. Indeed, there are certainly other examples that represent relationships in other non-traditional models (i.e. coordinated hunting in chimpanzees: 124; or tail-twining in titi monkeys: 125) that are not present in traditional models for endocrinological study, and the role of these specific behaviors can be contrasted among behaviors that are observed more generally (i.e., grooming).

## 5 Future directions

Oxytocin is clearly important in the expression of social behaviors, but many unanswered questions remain. More work is needed to understand the difference between how social behaviors impact oxytocin expression, and how changes in oxytocin levels influence behaviors. In particular, for the latter, it will be important to understand how typical changes as a result of natural social behavior versus induced changes as the result of some manipulation show similar or dissimilar effects. A second, related, question is to better understand the mechanism through which oxytocin is inducing these changes. Moreover, while it seems likely that there are effects directly on social behavior, it is also possible that the social behavior changes are mediated by a third mechanism, such as the anxiolytic effects of oxytocin (38, 70, 126). Finally, it will be important to further understand the interactions between these induced behaviors and individuals and relationships. It seems clear that oxytocin does not always unilaterally increase prosocial behaviors, but which factors are important for its different expression across different contexts, individuals, and relationships, and how different relationships affect responses to increases in these hormones, are unknown. Understanding the influence of development, social environment, or context will be key to characterizing these relationships. As an example, a recent study in female titi monkeys showed that dose sensitivity to exogenous oxytocin and vasopressin was related to previous affiliative bonds with their fathers (71), suggesting that early bonding behavior might influence later sensitivity to the hormones associated with social bonding.

Related to the above, we also need to develop better understanding of how oxytocin interacts with other hormones, like vasopressin, centrally, as well as how this interaction translates to peripheral endocrine relationships. There has been quite a lot of work with vasopressin in rodent models (for instance, a review of vasopressin research in prairie voles: 127), but that study priority has not yet emerged in primates, potentially due to another methodological trend – that previous methods used to study vasopressin are neurally focused rather than peripherally focused, which can be logistically and ethically challenging, especially in primates, and especially if those methods are terminal for the study subjects. However, as it may be difficult to direct measure, much less manipulate, central nervous activity in non-traditional species for which there is not yet a brain “atlas”, or for which typical neuroscience methods are precluded, it is important to first gain an understanding of how peripheral levels of these hormones might fluctuate in tandem or in synchrony in species for which we can then correlate this peripheral activity to direct neural activity (for instance, in prairie voles, for which a whole-brain atlas has been developed: 128). Understanding the central-peripheral relationship of oxytocin to other hormones in such species will then allow us to draw inferences about the functional significance of peripheral hormones in species and for ongoing behaviors for which it is difficult to measure central activity.

As alluded to above, much of the data from measurement studies comes from wild populations and is based on natural behavior. In contrast, most data from manipulation or induction studies come from captive populations, and while there are a few studies that explore its effect on natural behaviors in natural contexts (29), administration of oxytocin is typically done in more experimental contexts. One urgent need is to combine these approaches, to help us combine how oxytocin typically changes behavior across different social systems with how it can change behavior, and, again to better understand the difference between how behavior changes oxytocin expression, and how changes in oxytocin levels change behavior. For instance, currently it is extremely challenging to get oxytocin measurements in the field, but new biomarkers that are more logistically feasible in the field and more stable than oxytocin (but still are indicative of release of active molecules) will be essential. Co-peptin has proven to be a useful proxy biomarker for vasopressin, so it might be possible to find similar proxies for oxytocin (for instance, neurophysin-1: 129). Indeed, recent work has begun to explore if neurophysin-1, a part of the oxytocin protomolecule that serves as a carrier protein, might co-vary reliably with oxytocin in a way that would allow it to be used as a proxy for the more difficult to measure and more difficult to preserve oxytocin molecule. Additionally, if we can find other behaviors, such as fur rubbing, that allow us to exogenously manipulate endogenous release, we may be able to manipulate behavior in field studies that would then allow us to observe oxytocin’s effect on behavior in a natural context.

The study of oxytocin has expanded rapidly over the last several decades, allowing us to identify how a variety of factors related to species, social organization, and ecology are influencing responses. Although there are many open questions, these highlight the importance of this work for helping us understand how both endocrinological systems and social systems evolved. Despite the important key understanding of oxytocin that prior work has provided, we argue that expanding the focus to include species with social systems different from those that are currently the focus of oxytocin research (prairie voles, rats, rhesus macaques, marmosets) will be extremely helpful in this regard. Early work is promising, and we look forward to seeing what the next several decades bring.

## Author contributions

MS: Conceptualization, Project administration, Writing – original draft, Writing – review & editing. SB: Conceptualization, Supervision, Writing – original draft, Writing – review & editing.
